# Functional Benefits of (Modest) Alcohol Consumption

**DOI:** 10.1007/s40750-016-0058-4

**Published:** 2016-12-28

**Authors:** R. I. M. Dunbar, Jacques Launay, Rafael Wlodarski, Cole Robertson, Eiluned Pearce, James Carney, Pádraig MacCarron

**Affiliations:** Department of Experimental Psychology, University of Oxford, South Parks Rd, Oxford, OX1 4AA UK

**Keywords:** Social drinking, Social bonding, Happiness, Social networks, Conversational behaviour

## Abstract

Alcohol use has a long and ubiquitous history. Despite considerable research on the misuse of alcohol, no one has ever asked why it might have become universally adopted, although the conventional view assumes that its only benefit is hedonic. In contrast, we suggest that alcohol consumption was adopted because it has social benefits that relate both to health and social bonding. We combine data from a national survey with data from more detailed behavioural and observational studies to show that social drinkers have more friends on whom they can depend for emotional and other support, and feel more engaged with, and trusting of, their local community. Alcohol is known to trigger the endorphin system, and the social consumption of alcohol may thus have the same effect as the many other social activities such as laughter, singing and dancing that we use as a means of servicing and reinforcing social bonds.

## Introduction

The use of alcohol has deep historical roots, with archaeological evidence for the consumption of fermented beverages dating back at least 7000 years (Guasch-Jané et al. 2006; Sicard and Legras 2011; Dietrich et al. 2012). Although the reasons behind the prevalence of alcohol use are rarely investigated, there is an implicit assumption that its hedonic (physiological reward) and anxiolytic (reduction of anxiety or stress) properties are the main reasons for its universal use. However, alcohol also plays an important role in social contexts by reducing our social inhibitions, as well as being a potent trigger of the endorphin system (Froehlich 1997; Gianoulakis 2004; Herz 1997).

The link between alcohol and endorphin release is especially relevant, given the fact that the endorphin system lies at the heart of social bonding in human and nonhuman primates (Keverne et al. 1989; Panksepp 1999; Depue and Morrone-Strupinsky 2005; Dunbar 2010; Machin and Dunbar 2011; Nummenmaa et al. 2016). Not only does endorphin activation make us feel more relaxed (a natural property of opiates), but it also seems to ‘tune’ the immune system (Sarkar et al. 2012). Alcohol may also have indirect fitness benefits through the positive effect network size/composition has on health and survival that has been widely documented in both humans (House 2001; Kana’iaupuni et al. 2005; Kikusui et al. 2006; Charuvastra and Cloitre 2008; Reblin and Uchino 2008; Smith and Christakis 2008; Dominguez and Arford 2010; Holt-Lunstad et al. 2010; Pinquart and Duberstein 2010; Liu and Newschaffer 2011; Tilvis et al. 2012; Kim et al. 2016) and primates (Silk et al. 2003, 2009, 2010; Crockford et al. 2008; Wittig et al. 2008).

Perhaps because alcohol addiction has serious medical and social consequences, most research on alcohol use has focussed on the effects of over-consumption (Taylor et al. 2005; Easdon et al. 2005; Roerecke and Rehm 2010). Nonetheless, the fact that alcohol continues to be used, especially in social contexts, raises the question of why humans began to use it, and continue to use it so widely. We can identify two potential social benefits. One is that alcohol consumption enhances psychological wellbeing and, directly or indirectly, promotes the building of the close personal bonds that underpin social networks. In other words, it functions much like the many other behavioural mechanisms (including laughter, singing, dancing and storytelling: Dunbar et al. 2012; Tarr et al. 2015, 2016; Pearce et al. 2015; Dunbar et al. 2016) that are used to trigger the endorphin system so as to facilitate large-scale (i.e. communal as opposed to dyadic) social bonding. The other possibility is that alcohol in some way affects our social or cognitive skills in ways that allow us to function more effectively in social situations. While not all studies have shown such effects (see, for example, Dent et al. 1997), there is some evidence to suggest that there is an inverted U-shaped relationship between alcohol consumption and at least some forms of cognition (Christian et al. 1995; Hendrie et al. 1996; Launer et al. 1996; Elias et al. 1999; Galanis et al. 2000; Krahn et al. 2003; Britton et al. 2004; Lang et al. 2007; Sinforiani et al. 2011). This suggests that low-moderate levels of alcohol consumption might have beneficial effects on cognition, and that could include social cognition. For example, because alcohol consumption makes us more willing to take risks (Abrams et al. 2006; Sayette et al. 2012) and behave more competitively (Hopthrow et al. 2007), it might make us more willing to risk trying our luck with a prospective mate.

To explore this question, we combine data from a large national survey with data sampled in pubs and bars. Because our primary interest lies in the social aspects of alcohol consumption, we focus on the differences between individuals who have a regular drinking venue (a ‘local’), more casual drinkers who do not and non-drinkers. Because they provide very different social experiences, we also distinguish between smaller ‘community’-style pubs and city centre bars. At least in the UK, a community pub tends to be close to where its clientele live or work, such that regulars visit with sufficient frequency to know the staff and clientele on a personal basis; these pubs typically have a distinctly social ambience, with a smaller, quieter venue, are typically more beer-oriented than wine-oriented, and generally have lower per capita consumption (Jennings 2007). In contrast, city centre bars tend to be larger, have a clientele drawn from a much wider, more heterogeneous population, and commonly lack the community ambience of ‘locals’; their business plans typically focus on maximising alcohol sales. Since conversation is the principal means for creating and servicing friendships, we also undertook a study of the dynamics of conversation in these venues.

## Methodology

We combine data from three separate studies: a structured national survey, a questionnaire-based study of the clientele in a number of pubs and an observational study of conversational behaviour in pubs.

### Survey Sample

An online survey was commissioned from the polling agency YouGov by CAMRA (the Campaign for Real Ale). It asked about respondents’ drinking behaviour, their social networks and their wellbeing, using multiple-choice Likert-type responses. The survey was conducted over one week in November 2015 using a UK national randomly stratified sample of 2254 adults (aged 18+), representative of geographic region, gender and age. As well as background demographic information, the survey asked how often the respondent visited a pub, where they tended to drink and socialise, and whether they had a ‘local’. Respondents were then asked how socially connected they felt to their local community, using the *Inclusion-of-Other-in-Self* [IOS] rating scale (a 1–7 visual analogue scale, in which 1 indicates low connectedness and 7 indicates high connectedness) (adapted from Aron et al. 1992), how much they trusted people in general (on a 0–10 scale), and how many people they felt they could turn to for help if they needed to (an index of the size of their support network: Dunbar and Spoors 1995; Roberts et al. 2014). The survey also included measures of the respondents’ current sense of wellbeing using the following questions from the UK Office of National Statistics’ surveys, rated on a 0 (“not at all”) to 10 (“very”) scale:

- Overall, how satisfied are you with your life nowadays?
- Overall, how happy did you feel yesterday?
- Overall, how anxious did you feel yesterday?
- Overall, to what extent do you feel the things you do in your life are worthwhile?

When aggregating poll data, YouGov follows standard practice and applies a standard weighting (based on the Random Iterative Method, RIM: Deming and Stephan 1940) to account for variability in sampling across UK regions. RIM can result in a reduction of sample size, hence sample sizes cited in this paper are sometimes less than the full 2254 sample.

### Location-Based Studies

To study behaviour in pubs at first hand, we carried out two separate studies in pubs. The first focussed on social behaviour and cognition. We sampled 95 adults (31 women; mean age 34.1 ± 11.7 years, range 18–63; all native English speakers) in six pubs. Four were community pubs and two were large city-centre bars. Participants were invited to take part in a set of short tasks on handheld devices. They were reimbursed £5 for their time. After they had completed the tasks, they were given a breathalyser test to determine their alcohol consumption. To make sure that accurate readings were taken, they were asked not to drink while carrying out the questionnaire tasks. Only 13% of the individuals sampled exceeded the UK legal blood alcohol limit for drink-driving, and only three (3.1%) had an alcohol level that was more than twice the drink-drive limit.

The first part of the questionnaire asked about their drinking habits: how frequently they visited pubs, their weekly alcohol consumption, what they typically drank, whether they had a ‘local’, and whether they had consumed any alcohol before coming to the pub. Next, participants were asked to rate how well-integrated into their local community they felt themselves to be (using the same IOS task as used in the national survey); a note was made of the size of the social group they were in. Finally, they were given two picture-based tasks designed to assess their social appraisal of strangers.

In the first of these tasks, participants were shown 20 photos (10 male and 10 female) of standardised faces from the Chicago Face Database (Ma, Correll & Wittenbrink 2015) and asked to rate them for trustworthiness, approachability and attractiveness on a 7-point Likert scale (1 = ‘Not at all’, 7 = ‘Extremely’). The faces were sampled randomly from the database since we were not interested in actual attractiveness, but how individuals perceived faces. This task assessed people’s propensity to make socially relevant appraisals of strangers in ways that might influence their willingness to initiate interaction, thus indicating whether their social judgments of others are influenced by alcohol. Participants were also presented with a validated measure of social cognition (the Reading the Mind in the Eyes task, RMET: Baron-Cohen and Wheelwright 2001). For the RMET, subjects view a series of photos showing just the eye region of faces, and a choice of four words describing different emotions. They were asked to choose which word best described the emotion displayed in the photo. This task measures the ability to identify emotions from other people’s faces, a capacity that underlies empathy (putting yourself in someone else’s shoes) and hence mindreading (or mentalising) – the ability to understand someone else’s thoughts (a key social skill) (Baron-Cohen and Wheelwright 2001). Mentalising is a core process central to human sociality.

Since conversation is at the heart of sociality, we undertook a second observational study focussed on conversational behaviour in seven different pubs (five large city-centre bars, and two local community pubs). The Animal Behaviour Pro app was used on iPhones and iPads to record the conversational behaviour of 65 focal individuals for 20 min each (total sample time was 21.6 h). Subjects were not approached and were unaware that they were being observed. A subject from an obviously interacting group was randomly selected, their gender recorded as well as that of anyone they were sitting with at the time. For the next 20 min, the researcher recorded who the focal individual was speaking or listening to, as well as whether anyone arrived or left their group. The researchers used eye contact and speaking as criteria for who was engaged with their focal individual. The observer also noted when individuals were not paying attention to the conversation (defined by behaviours such as not talking, sitting in silence, staring off elsewhere round the room, waiting to be served at bar, or on their phone). At the end of each sample period, the researcher randomly selected a new focal individual from another group. At any single venue, researchers conducted roughly 6 samples (representing two hours of data collection), or as many as there were separate groups in the pub. Two people were never sampled from the same conversation group. A total of 283 people (135 males), including the 65 focal individuals, were recorded as being involved in the sample conversations.

## Results

### Survey Data

**Fig. 1:**
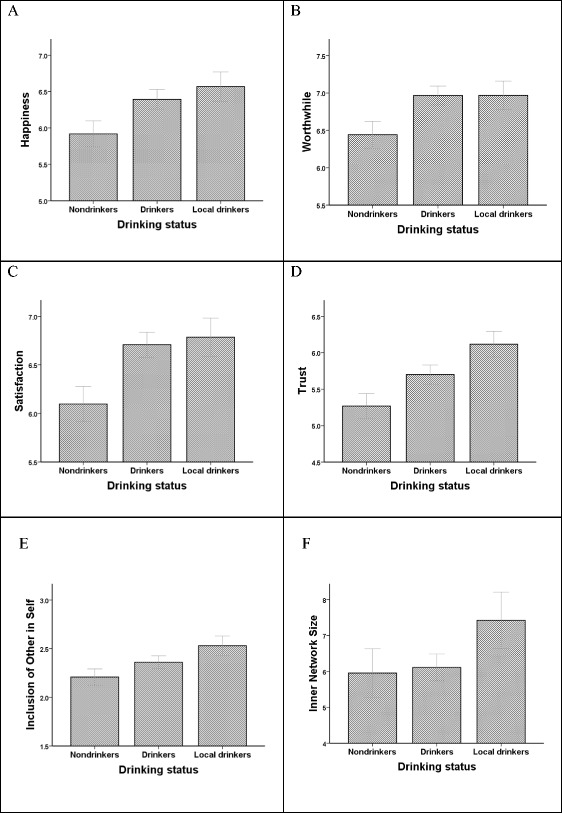
Mean (±2 se) ratings of **a** happiness on the previous day, **b** feelings of worthwhileness, **c** satisfaction with life, **d** trust in other people, **e** feeling connected to the local community (indexed as the Inclusion-of-Other-in-Self, IOS, scale: 1 = not at all connected, 7 = very connected) and (f) number of intimate friends (on whom one can rely for social, emotional and financial support), as a function of whether or not participants drink alcohol regularly and whether or not they have a ‘local’ pub they visit regularly. Source: YouGov national UK poll

Of the 2254 respondents, 708 declared that they were non-drinkers or rare drinkers, 946 said they regularly drank alcohol but had no ‘local’ they visited regularly, and 447 that they had a ‘local’ (153 respondents did not answer the question). Figure 1 plots the responses to the main questions as a function of these three groupings. The differences across the three categories in personal happiness, perceived worthwhileness of life and satisfaction with life were all significant (Fig. 1a: Kruskal-Wallis ANOVA, *χ* ^2^ = 25.88, *df* = 2, *p* < 0.0001; Fig. 1b: *χ* ^2^ = 12.63, *df* = 2, *p* = 0.002; Fig. 1c: *χ* ^2^ = 36.20, *df* = 2, *p* < 0.0001): in each case, pairwise comparisons reveal a significant difference between non-drinkers and drinkers (*p* < 0.05), but no differences between regular drinkers with and without a ‘local’. Scores for trust in others and engagement with the local community (IOS) also differed significantly between categories (Fig. 1d: *χ* ^2^ = 39.18, *df* = 2, *p* < 0.0001; Fig. 1e: *χ* ^2^ = 30.50, *df* = 2, *p* < 0.0001), with all pairwise differences significant (*p* < 0.01). The number of intimate friends (support clique) averaged 6.29 ± 7.69SD, which is on the high side but within the range of variation observed in other samples (Dunbar et al. 1995; Sutcliffe et al. 2012; Burton-Chellew and Dunbar 2015). The differences between the three categories in support clique size are significant (Fig. 1f: *χ* ^2^ = 33.40, *df* = 2, *p* < 0.0001): the difference between non-drinkers and drinkers was highly significant, but regular drinkers were only marginally significantly different from those who had a ‘local’. In contrast, the differences between categories in the rated level of personal anxiety were not significant (data not shown: *χ* ^2^ = 1.27, *df* = 2, *p* = 0.531).

**Fig. 2:**
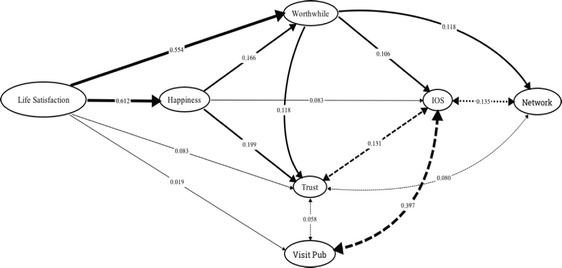
Path analysis of the main variables in the UK national poll dataset (*N* = 2254 participants). Arrows indicate significant (*p* < 0.05) standardized ßs (given by the numbers against the arrows) from multiple regressions with each variable in turn as the dependent variable. All variables are scaled data. Single headed arrows: relationships in which the ß for the indicated direction is at least twice that for the converse direction. Double headed arrows and dashed lines: significant relationships of approximately equal weight (the larger ß is indicated in each case). Strength of the relationship is indicated by line weight

In order to evaluate the most likely causal relationships between these variables, we ran a path analysis. We excluded anxiety from this analysis, since it is unrelated to pub use and correlated (negatively) only with happiness (*r* = −0.419, *N* = 2254, *p* < 0.0001) and life satisfaction (*r* = −0.339, *p* < 0.0001), but included the frequency with which participants stated that they visited pubs (on a 5-point Likert scale) as the key variable of interest. Figure 2 shows all the significant partial *ß*s, with a number of these being explicitly one-directional. These suggest a causal sequence that runs from satisfaction with life to both happiness and increased frequency of pub visits, which between them independently influence one’s sense that life is worthwhile and the level of trust in, and connection with, the local community (the latter indexed by IOS), which in turn influence the number of intimate friends one has.

Taken together, these data suggest that there are benefits to be derived directly from drinking alcohol, especially in relaxed social environments. These effects are clearly involved in a complex feedback process, and it is clear from the path analysis that certain types of people (those who feel more satisfied with their lives) are more likely to visit pubs and benefit from these effects.

### In-Pub Behavioural Data

Subjects sampled in the different venues did not differ significantly in blood alcohol level: a 2 × 2 × 2 ANOVA with customer type (‘regulars’ vs casuals: i.e. drinking in their ‘local’ or not), venue (community pub vs city centre bar) and sex as factors was not significant (overall model *F*_3,90_ = 1.96, *p* = 0.126). Only sex came marginally close to significance, with women having consumed less than men on average (*F*_1,90_ = 3.39, *p* = 0.069).

**Fig. 3:**
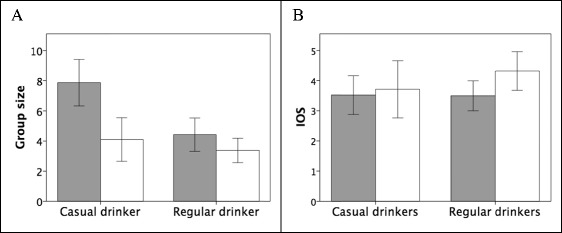
**a** Mean (±2se) conversation group size and **b** mean (±2se) feeling of connectedness to the local community (indexed as the IOS: 1 = low, 7 = high) for individuals who were casual drinkers versus regular drinkers in the sampled venues, differentiated by whether the venue was a community pub (*shaded bars*) or a city centre bar (*open bars*). Source: pub behavioural sample

Those who declared that they were in their ‘local’ (‘regulars’) or who were in community pubs were in significantly smaller social groups than those who were casual visitors in city centre bars (Fig. 3a: regulars, mean group size 3.94 ± 2.78SD vs 6.73 ± 3.74 for casuals; community pubs, mean = 3.56 ± 2.20 vs 5.84 ± 3.77 in city bars). 2 × 2 × 2 ANOVA yielded a significant model (*F*_3,91_ = 9.00, *p* < 0.001), with significant individual effects for customer type (*F*_1,91_ = 13.51, *p* < 0.001) and pub type (*F*_1,91_ = 8.55, *p* = 0.004), but not for sex (*F*_1,91_ = 0.03, *p* = 0.855). Note that those attending their ‘local’ and those in community pubs were in conversation-sized groups (which typically have a maximum size of 4: Dunbar et al. 1995; Dezecache and Dunbar 2012; Dunbar 2016; Krems et al. 2016; Dahmardeh and Dunbar 2017), whereas casual customers and those in city centre bars were typically in parties that were larger than the normative limit for conversations.

Figure 3b plots how integrated into their local community people felt themselves to be (indexed by the IOS scale). Subjects in smaller, community-type pubs were more likely to feel that they were a member of their community than those in larger city centre bars. However, the 2 × 2 × 2 ANOVA model with venue, customer and sex as predictors was not significant (*F*_3,79_ = 1.51, *p* = 0.219), with location the only factor that was (marginally) significant (*F*_1,79_ = 3.71, *p* = 0.058).

Participants were asked to rate the approachability, trustworthiness and attractiveness of a set of photographs of male and female faces; they also took the Reading-the-Mind-in-the-Eyes (RMET) test. For all four indices, there was no relationship between mean score and blood alcohol level (linear or quadratic fits: *F*_1,86_ ≤ 1.406, *p* ≥ 0.226). Nor were any of the linear or quadratic regressions significant when split by type of venue or gender.

### Conversational Dynamics

**Fig. 4:**
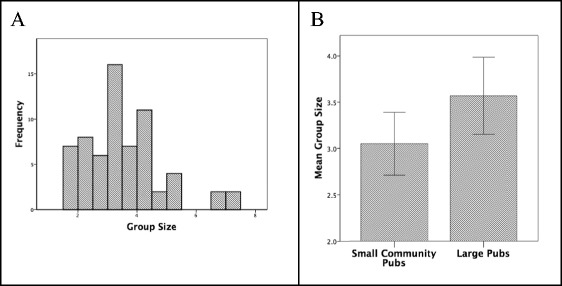
**a** Distribution of mean weighted conversation group sizes in pubs. **b** Mean (±2se) weighted conversation group size in small community pubs (‘locals’) versus larger city centre bars. Weighted group size is group size weighted by the length of time the conversation was at a given group size, and so takes account of the changes in size as individuals join and leave a conversation. Source: pub conversational behaviour sample

Across all venues, the average size of conversations was 3.44 ± 1.33 *SD* (Fig. 4a). This is again in close agreement with previous samples of conversation group size. Although the average conversation group size was larger in city centre bars than in community pubs (Fig. 4b), the difference was not significant. However, conversations in city centre bars had a significantly greater range than those in community pubs (Levene’s test for homogeneity of variances: *F*_1,64_ = 8.002, *p* = 0.006, *n* = 65 groups).

**Fig. 5:**
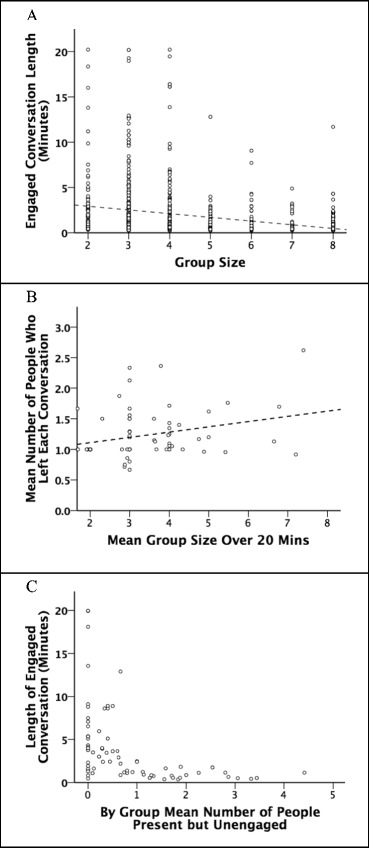
**a** Conversation duration plotted against conversation group size. **b** Mean number of people who left a conversation as a function of the number of people in the group at the time they left. **c** Duration of a conversation as a function of the number of people in the conversation who were not engaged with (i.e. paying attention to) the speaker. Source: pub conversational behaviour sample

The size of a social group had significant consequences for its dynamics. Irrespective of venue, conversations became more fragmented as the size of the group increased, with continuous stretches of conversation being shorter (Fig. 5a: Pearson’s *r* = −0.423, *p* < 0.001, *n* = 65 groups) and more people dropping out of the conversation as group size increased (Fig. 5b: Pearson’s *r* = 0.285, *p* = 0.022, *n* = 65 groups). Unbroken chains of conversation were also significantly shorter in proportion to the number of individuals in the group that were not paying attention to the speaker (Fig. 5c: Pearson’s *r* = −0.449, *p* < 0.001, *n* = 65 groups).

**Fig. 6:**
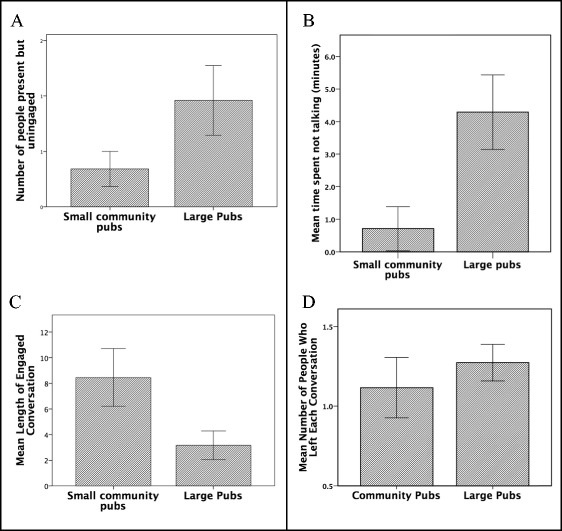
**a** Mean (±2se) number of people present but not actively involved in a conversation (talking or listening) in small community pubs and large pubs/bars. **b** Mean (±2se) time spent not talking in conversations in small community pubs and large pubs/bars. **c** Mean (±2se) length of conversations in small community pubs and large pubs/bars. **d** Mean (±2se) number of people who dropped out of each conversation in small community pubs and large pubs/bars. Source: pub conversational behaviour sample

A two-way ANOVA indicated that the proportion of people who were not engaged with (i.e. paying attention to) a conversation they were physically part of was significantly higher in city centre bars than in community pubs (Fig. 6a: *F*_*1*,64_ = 4.85, *p* = 0.031); in fact, people in city centre bars spent significantly more time *not* taking part in the conversation they were associated with (Fig. 6b: *F*_*1*,64_ = 12.15, *p* < 0.001). No one was ever recorded checking their phone in any of the samples in a small community pub, but in large city centre bars people often did so. Overall, the total time people spent on their phones was significantly positively correlated with total time spent not talking (Pearson correlation: *r* = 0.311, *p* = 0.012). Consequently, conversations lasted significantly longer in community pubs than they did in city centre bars (Fig. 6c: *F*_*1*,64_ = 20.47, *p* < 0.001). There was a tendency for people to drop out of conversations more often in the latter type of venue, but the difference was not statistically significant (Fig. 6d: *F*_*1*,64_ = 2.01, *p* < 0.161).

In sum, conversations in community-type pubs were longer, more focussed and less liable to fragmentation than those in city centre bars.

## Discussion

The aim of this study was to ask whether there was any evidence that alcohol consumption has social benefits beyond a simple hedonic ‘high’ or anxiolytic effect. Because alcohol triggers an endorphin response, we hypothesised that it might increase the degree of social bonding (feelings of social closeness: see Dunbar and Shultz 2010; Roberts et al. 2014) and this might have implications for how happy and socially engaged people become. The evolutionary significance of this lies in the fact that our social networks provide us with the single most important buffer against mental and physical illness (House 2001; Fowler & Christakis 2008; Holt-Lunstad et al. 2010; Tilvis et al. 2012; Kim et al. 2016). We asked whether the frequency of social alcohol consumption (indexed by the frequency of drinking in pubs) or the type of venue (‘locals’ vs bars) influenced people’s social experiences and their wellbeing.

The survey data suggest that respondents who have a ‘local’ that they visit on a regular basis are more socially engaged, feel more contented in their lives, and are more likely to trust other members of their community. On some, but not all of our social measures, those who drink ‘casually’ were more socially engaged than those who didn’t drink at all, suggesting that there are independent effects due to being a drinker and having a regular drinking venue. Overall, the number of close friends that people have (those on whom one can count for support in times of crisis, often known as the ‘support clique’) is of the same magnitude as has been found in previous studies (present study: 6–7; previous studies: 4–6: Sutcliffe et al. 2012). However, those who did not have a ‘local’ had significantly smaller social networks and felt less engaged with, and trusting of, the communities within which they were embedded. The path analysis suggested that feeling satisfied with life and how often one visits a pub both independently influence a set of variables associated with happiness and trust in others, which in turn influence engagement with the community and personal network size.

The results of the pub behavioural study corroborated the findings from the national survey: people drinking in community pubs (or ‘locals’) felt significantly more engaged with their local community than those drinking in city centre bars. More importantly, those in ‘locals’ were more likely to be in ‘conversational’ sized groups, whereas those drinking in city centre bars were in groups that significantly exceeded not only natural conversation group size but also the typical size of the support clique. This difference in social environment may be expected to have significant effects on the formation and maintenance of social bonds. In large city centre venues, people were much less engaged with each other, moving rapidly from one brief conversation to another. As a result, they have less time to get to know their social companions or establish relationships with them. We interpret this as being at least part of the explanation for the fact that those who do not have a ‘local’ they visit regularly feel less engaged with their community, feel less satisfied with life and have smaller support networks. This may have wider implications because the size of support networks (cliques) scales up to predict the size of the extended social network (or active network) (Zhou et al. 2005; Sutcliffe et al. 2012).

It is always possible that these differences in behaviour might be due to personality differences. Extraverts, for example, have larger social networks than introverts, although the average quality of their relationships is typically weaker as a result (Roberts et al. 2008; Pollet et al. 2011). We did not include measures of personality because we did not want to overburden our participants, and hence we cannot evaluate this possibility here. However, we take the view that any relationship between particular personality dimensions and our dependent measures (happiness, social engagement) is likely to be mediated by the role of social drinking, or at least that the two effects are independent and additive. This remains to be tested, however.

Although there is considerable evidence that moderate alcohol consumption can enhance some aspects of cognition, including memory, mental arithmetic and inhibition, even though excessive consumption inevitably has deleterious effects (Christian et al. 1995; Henrie et al. 1996; Launer et al. 1996; Elias et al. 1999; Galanis et al. 2000; Krahn et al. 2003; Schreckenberger et al. 2004; Easdon et al. 2005; Lang et al. 2007; Sinforiani et al. 2011), we could detect no effect on people’s assessment of strangers’ approachability, trustworthiness or attractiveness on the basis of facial cues, at least within the modest range of alcohol consumption that we sampled. There is an important contrast between most laboratory studies of the effects of alcohol, where subjects are required to consume large doses of alcohol (in some cases, by intravenous injection) in artificial settings, and our real-world observational study involving mostly moderate social drinking. The lack of any effects of social alcohol consumption on social cognition and the positive findings on the more general aspects of life satisfaction and social engagement outside the immediate pub environment suggests that the role of alcohol is more likely associated with the maintenance of existing relationships than with the initiation of new ones with strangers.

Because the endorphin system is central to social bonding in primates (including humans) (Keverne et al. 1989; Panksepp 1999; Depue and Morrone-Strupinsky 2005; Dunbar 2010; Machin and Dunbar 2011; Nummenmaa et al. 2016) and seems to have a direct effect on the body’s capacity to resist endogenous and exogenous disease threats (Sarkar et al. 2012; Kim et al. 2016), anything that triggers the endorphin system is likely to have been adopted once discovered. This is not, of course, to suggest that excessive alcohol consumption does not have serious health consequences.

Aside from direct health benefits that might arise from up-regulating the endorphin system, the principal benefit of the social consumption of alcohol may thus be that it acts much like the many other endorphin-stimulating activities that we use for social and community bonding (notably laughter, singing, dancing, and even storytelling: Dunbar et al. 2012; Tarr et al. 2015, 2016; Pearce et al. 2015; Dunbar et al. 2016). This is not to suggest that alcohol consumption is an adaptation in the formal biological sense, but rather that we discovered how it could be used to trigger a mechanism (the endorphin system) that is an adaptation for social bonding. Indeed, there is now a widespread view among archaeologists that cereal cultivation was first started in order to brew beer rather than to provide food (Dietrich et al. 2012; Hayden et al. 2013). We suggest that, like these other social bonding activities, the consumption of alcohol, once it had been discovered, came to be adopted as part of the complex set of activities and rituals associated with bonding our (by monkey and ape standards) large social communities.
